# Cathelicidin Modulates Vascular Smooth Muscle Cell Phenotypic Switching through ROS/IL-6 Pathway

**DOI:** 10.3390/antiox9060491

**Published:** 2020-06-05

**Authors:** Xiaoliang Dong, Di Wu, Yihan Zhang, Lingling Jia, Xiaohua Pan, Jia Sun, Li-Long Pan

**Affiliations:** 1 Wuxi School of Medicine, Jiangnan University, Wuxi 214122, Jiangsu, China; 8201901030@jiangnan.edu.cn (X.D.); 6192807015@stu.jiangnan.edu.cn (D.W.); jialingling@jiangnan.edu.cn (L.J.); 2 School of Food Science and Technology, Jiangnan University, Wuxi 214122, Jiangsu, China; 1012170824@stu.jiangnan.edu.cn (Y.Z.); panxiaohuacaas@163.com (X.P.); 3 State Key Laboratory of Food Science and Technology, Jiangnan University, Wuxi 214122, Jiangsu, China

**Keywords:** atherosclerosis, Cathelicidin-related antimicrobial peptides, vascular smooth muscle cell, phenotypic transformation, oxidative stress

## Abstract

Vascular smooth muscle cells (VSMC) are stromal cells of the blood vessels and their differentiation is thought to be essential during atherosclerosis. Cathelicidin-related antimicrobial peptides (CRAMP) are suggested to play a role in the development of atherosclerosis. Even so, the relationship of CRAMP and VSMC remains unclear. The present study was to determine whether CRAMP regulates VSMC phenotypic transformation and underlying mechanisms. We demonstrated that CRAMP could reverse platelet-derived growth factor-BB (PDGF-BB)-induced VSMC phenotypic transformation, evidencing by increasing α-smooth muscle actin (α-SMA), smooth muscle 22α (SM22α) and decreasing of proliferation and migration. Further studies showed that CRAMP inhibited nuclear factor κB (NF-κB)-induced autocrine of interleukin-6 (IL-6), which further activated of janus kinase 2 (JAK2)/signal transducer and activator 3 (STAT3). Meanwhile, our data showed that CRAMP can significantly inhibit PDGF-BB enhanced intracellular reactive oxygen species (ROS) level which further affected the NF-κB signaling pathway, indicating that CRAMP can regulate the phenotypic transformation of VSMC by regulating oxidative stress. These results indicated that CRAMP regulated the differentiation of VSMC by inhibiting ROS-mediated IL-6 autocrine, suggesting that targeting CRAMP is a potential avenue for regulating the differentiation of VSMC and treatment of atherosclerosis.

## 1. Introduction

Cardiovascular disease, the world’s leading cause of death, claiming an estimated 17.9 million lives each year, is a group of heart and vascular diseases such as atherosclerosis [1]. In the study of the development of atherosclerosis, more and more emphasis has been placed on the phenotypic transformation of vascular smooth muscle cells (VSMC) [2]. VSMC phenotypic modulation from a contractile to a synthetic phenotype in vessel walls, triggered by harmful microenvironmental stimuli and followed by VSMC migration and proliferation, is critical for the development of proliferative vascular disease [3,4,5]. Phenotypic modulation of VSMC from a quiescent and contractile type to synthetic phenotype is an important step in the development of several pathophysiological processes such as atherosclerosis, restenosis and vascular remodeling [6,7]. In VSMC, this is characterized by an increase in proliferation, migration and extracellular matrix protein production and a decrease in the expression of cytoskeletal and contractile proteins such as α-SMA, calponin and myosin heavy chain [7]. Imbalanced VSMC plasticity results in maladaptive phenotype alterations that ultimately lead to progression of a variety of VSMC-driven vascular diseases [8]. Therefore, regulating VSMC phenotype transformation may be a means of treating cardiovascular disease.

With the development of research, more and more factors are considered to affect the phenotypic transformation of vascular smooth muscle. The stimuli triggering the phenotypic modulation include injury, mechanical force, oxidative stress, intracellular and extracellular microenvironment and molecules, and cell-cell and cell-matrix interactions [8,9,10,11,12]. Several studies have demonstrated that oxidative stress plays an important role in the pathogenesis of cardiovascular alterations observed in diabetic patients [13,14,15]. Oxidative stress occurs when the concentrations of reactive oxygen species (ROS) exceed those of antioxidant neutralizing species, such as nicotinamide adenine dinucleotide phosphate (NADPH) and glutathione. ROS is a heterogeneous population of molecules including free radicals, such as hydroxyl, superoxide, peroxyl, and hydroperoxyl, and non-radical species, as hydrogen peroxide and hydrochloric acid [16,17]. The generation of intracellular ROS can subsequently activates the redox sensitive transcription factor nuclear factor κB (NF-κB), which modulates the expression of a variety of genes associated with inflammation and atherosclerosis, including interleukin-6 (IL-6) [18,19]. The elucidation of the mechanisms regulating the VSMC phenotypic modulation is of utmost importance for providing insight toward a better understanding of the occurrence and development of vascular disease and for developing a better therapeutic strategy.

Antimicrobial peptides (AMPs) are short cationic molecules that are components of innate immunity and can defend against invading pathogens [20]. AMPs have been found to exert different functions in the host immune protection, such as antitumor, wound healing and anti-obesity [21]. Among AMPs, the role of the cathelicidins (human LL-37 and mouse CRAMP) has been particularly documented in autoimmune diseases such as atherosclerosis, small-vessel vasculitis, systemic lupus erythematous, and psoriasis [22,23,24,25]. Although the antimicrobial peptide Cramp/LL37 can play a role in atherosclerosis by activating autoimmune [22], it is not known whether it can play a role through directly targeting VSMC. Studies showed that Cathelicidin-WA (CWA), a novel cathelicidin peptide from snakes, could markedly reduce E. coli K88-induced ROS accumulation [26] and mitigate Lipopolysaccharide (LPS)-induced ROS accumulation in macrophages and in mice [27]. Interestingly, LL-37 treatment induced several times greater ROS production compared to controls in mast cells [28]. Therefore, the role of CRAMP in ROS regulation needs to be further determined.

In this study, we discovered that CRAMP could modulate phenotype in VSMC. Furthermore, we found that CRAMP alleviated platelet-derived growth factor-BB (PDGF-BB)-induced proliferation, migration and phenotypic transformation of VSMC through modulating ROS/NF-κB, which was dependent on IL-6 autocrine. These findings contribute to our understanding of the mechanism of Cathelicidin peptides on regulating atherosclerosis.

## 2. Materials and Methods

### 2.1. Reagents and Antibodies

The antimicrobial peptide CRAMP (SP-CRPL-5) was purchased from Innovagen, Sweden. PDGF-BB (CYT-740) was obtained from ProSpecbio (Ness-Ziona, Israel), dissolved in sterile deionized water, and stored at −20 °C. Anti-α-smooth muscle actin (α-SMA), anti-smooth muscle 22α (SM22α) and anti-interleukin-6 (IL-6) (Neutralizing) (ab9324) antibody were purchased from Abcam technology (Cambridge, UK). Anti-signal transducer and activator (STAT3), anti-phospho-STAT3 (Tyr705), anti-extracellular signal-regulated kinase 1/2 (ERK1/2) and anti-phospho-ERK1/2 (Thr202/Tyr204), anti-phospho-inhibitor of nuclear factor kappa-B kinase (IKKα/β) (Ser176/180) (#2697) were purchased from Cell Signaling Technology Inc. Anti-glyceraldehyde-3-phosphate dehydrogenase (GAPDH) (AP0063) and Goat anti-Rabbit IgG (H&L)-horseradish peroxidase (HRP) (BS12478) antibodies were obtained from Bioworld, USA. Anti-IL-6 (A0286), anti-phospho-janus kinase 2 (JAK2) (Y1007/1008) (AP0531), anti-β-actin (AC026), anti-IκBα (A19714), anti-phospho-inhibitor of nuclear factor kappa-B (IκBα) (AP0707), anti-NF-kB p65 (A2547) antibodies were purchased from ABclonal. Anti-JAK2 (17670-1-AP), anti-Histone-H3 (17168-1-AP), anti-IKK (15649-1-AP), anti-NADPH oxidase (NOX)1 (17772-1-AP), anti-NOX2 (19013-1-AP), anti-NOX4 (14347-1-AP) were purchased from Proteintech, Manchester, UK.

### 2.2. Primary VSMC Culture

VSMC were harvested from normal rat aortas using the explant technique. The VSMC were cultured routinely in Dulbecco’s modified Eagle’s medium (DMEM; Hyclone Laboratories Inc., Logan, UT, USA) containing 10% fetal bovine serum (FBS), supplemented with penicillin (100 U/mL) and streptomycin (100 μg/mL) at 37 °C with a humidified atmosphere of 5% CO_2_. The primary VSMC were identified using smooth muscle α-actin antibody. For all experiments, VSMC (2–5 passages) were used following by quiescence for 12 h.

### 2.3. Cell Viability Assay

Cells were seeded into 96-well plates and allowed to adhere for 24 h. After being treated with different doses of CRAMP for 48 h, cells were subjected to viability detection by using the 3-(4,5-dimethylthiazol-2-yl)-2,5-diphenyl tetrazolium bromide (MTT) assay kit (Sigma-Aldrich, St. Louis, MO, USA) according to the manufacturer’s specifications. In brief, cells in each well were incubated with 10 μL MTT working solution at 37 °C for 4 h. The absorbance of each well at 490 nm was measured using an Epoch Microplate Reader (BIO-TEK, Winooski, VT, USA).

### 2.4. Western Blot

Tissues and cells were lysed by using lysis buffer and centrifuged at 13,000× *g*, 4 °C. Samples incubated with a sodium dodecyl sulfate (SDS) sample loading buffer were heated on the boiling water bath for 5 min, then subjected to 12% SDS-polyacrylamidegel (PAGE), and transferred onto polyvinylidene fluoride (PVDF) membranes. After being blocked in 5% fat-free milk at room temperature (RT) for 1 h, membranes were incubated overnight at 4 °C with primary antibodies, followed by HRP-conjugated secondary antibodies for 1 h at RT. Finally, membrane-bound antibodies were detected using a chemiluminescence reagent. The total protein content of loading was monitored by reprobing the same blots with loading control.

### 2.5. Animal Experiments

C57BL/6 mice were purchased from JOINN Laboratories (Suzhou). CRAMP knockout C57BL/6 mice were preserved in our laboratory. All animal experimental procedures were approved by the Jiangnan University Experimental Animal Management and Animal Welfare Ethics Committee (IACUC Issue Number: JN. No20191015c0401218). Mice were raised under conventional controlled conditions (22 °C, 55% humidity and day-night rhythm) and had free access to a standard diet and tap water. All mice were allowed to acclimate to these conditions for at least 2 days before inclusion in experiments.

### 2.6. Proliferation Assay

Thus, 5-Ethynyl-2′-Deoxyuridine (EdU) was used to detect the proliferation of VSMC cells according to the Cell-Light^TM^ EdU Apollo^®^488 In Vitro Imaging Kit (Guangzhou Ribobio, China) instructions. Briefly, cells were cultured in 96-well plates, after treatment, EdU (100 mM, 100 μL/well) was added and incubated for 12 h. Following, VSMC cells were fixed with 4% paraformaldehyde. After 30 min, 1× Apollo^®^ staining reaction liquid was added (100 μL/well), and cells were incubated for 30 min at room temperature; After 10 min of permeabilization with 0.5% Triton X-100, the cells were stained with 1× Hoechst 33,342 (100 μL/well) for 30 min. The ratio of EdU-positive cells (EdU-stained cells/Hoechst-stained cells × 100%) was determined using a fluorescence microscope (Nikon Eclipse Ti-S, Tokyo, Japan).

### 2.7. Enzyme-Linked Immunosorbent Assay (ELISA) Assay

IL-6 ELISA detection kit (SBJ-M0657) were purchased from SenBeiJia Biological Technology Co., Ltd. (Nanjing, Jiangsu, China). Labeled antibodies and the biotin were co-incubated with the test samples. The optical density (OD) values of the samples were detected using a BioTek microplate reader. Standard curves were plotted according to standard OD values, and test sample concentrations were calculated from the standard curve.

### 2.8. ROS Detection

ROS was detected by using the commercial assay kit. Briefly, cell extracts were incubated with ROS specific dye, 2, 7-dichlorofuorescin diacetate (DCFH-DA), at 37 °C for 30 min, and then were centrifuged, washed and suspended in PBS. ROS were detected by using Epoch Microplate Reader (BIO-TEK, VT, USA) at 525 nm and Fluorescence microscope. Dihydroethidine hydrochloride (5 μM, Molecular Probes) was topically applied to the freshly cut frozen aortic sections (10 μm) for 30 min at 37 °C to reveal the presence of ROS as red fluorescence (585 nm) by Fluorescence microscopy.

### 2.9. Statistical Analysis

All statistical analysis was carried out by using GraphPad Prism software. Error bars for in vitro and in vivo analysis represent the standard deviation among intra-class data collected from more than 3 independent experiments. Data were analyzed by using analysis of variance (ANOVA). Statistical significance was determined using unpaired Student’s two-tailed t-test for two data sets and using a one-way ANOVA and followed by least significance difference multiple comparison tests. Statistical significance was defined as * *p* < 0.05; ** *p* < 0.01; *** *p* < 0.001.

## 3. Results

### 3.1. CRAMP Inhibits PDGF-BB-Induced VSMC Phenotypic Transformation, Proliferation and Migration

**Figure 1:**
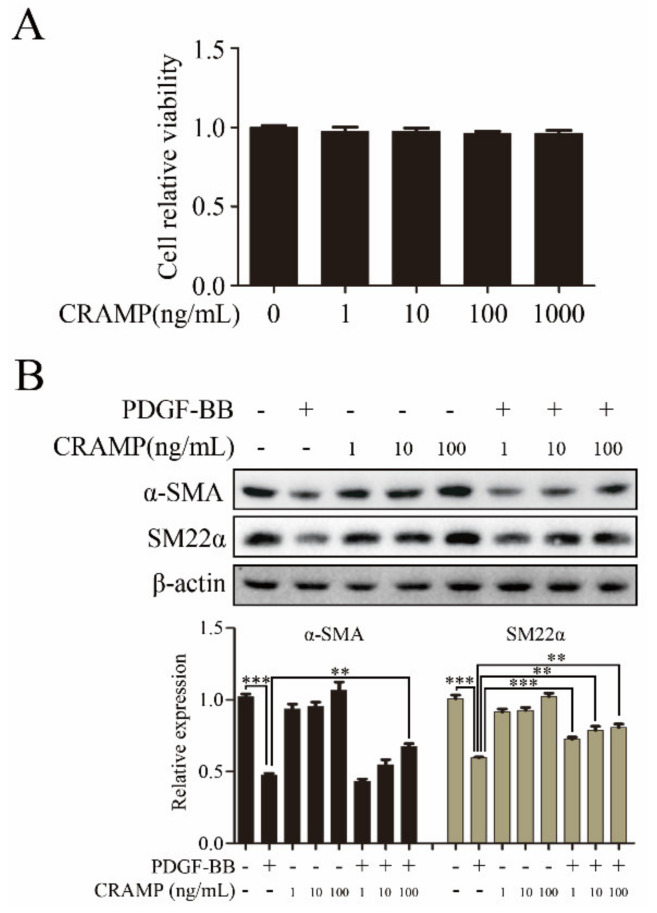
Cathelicidin-related antimicrobial peptides (CRAMP) inhibits platelet-derived growth factor-BB (PDGF-BB)-induced vascular smooth muscle cells (VSMC) Phenotypic transformation. (**A**) Measurement of changes in cell viability of VSMC after 48 h incubation with a range of concentrations (0, 1, 10, 100 and 1000 ng/mL) of CRAMP. (**B**) VSMC were pretreated with CRAMP (100 ng/mL) for 2 h and then stimulated with PDGF-BB (20 ng/mL) for 24 h followed by immunoblotting with α-SMA and SM22α antibodies. Data of 3 independent experiments is presented as mean ± SEM. ** *p* < 0.01; *** *p* < 0.001 compared with control.

VSMC phenotypic change to dedifferentiated state was a key step in arterial neointimal hyperplasia during the formation of restenosis [29]. To investigate the function of CRAMP on VSMC phenotypic transformation, we first detected the cytotoxity of CRAMP on VSMC. The MTT assay showed that CRAMP have almost no effects on VSMC at the maximum dose at 1000 ng/mL (Figure 1A). Furthermore, the western blot results showed that CRAMP concentration-dependently reversed PDGF-BB-mediated the decrease of α-SMA and SM22α expression (Figure 1B). These results suggested that CRAMP could inhibit PDGF-BB-induced VSMC phenotypic transformation.

**Figure 2:**
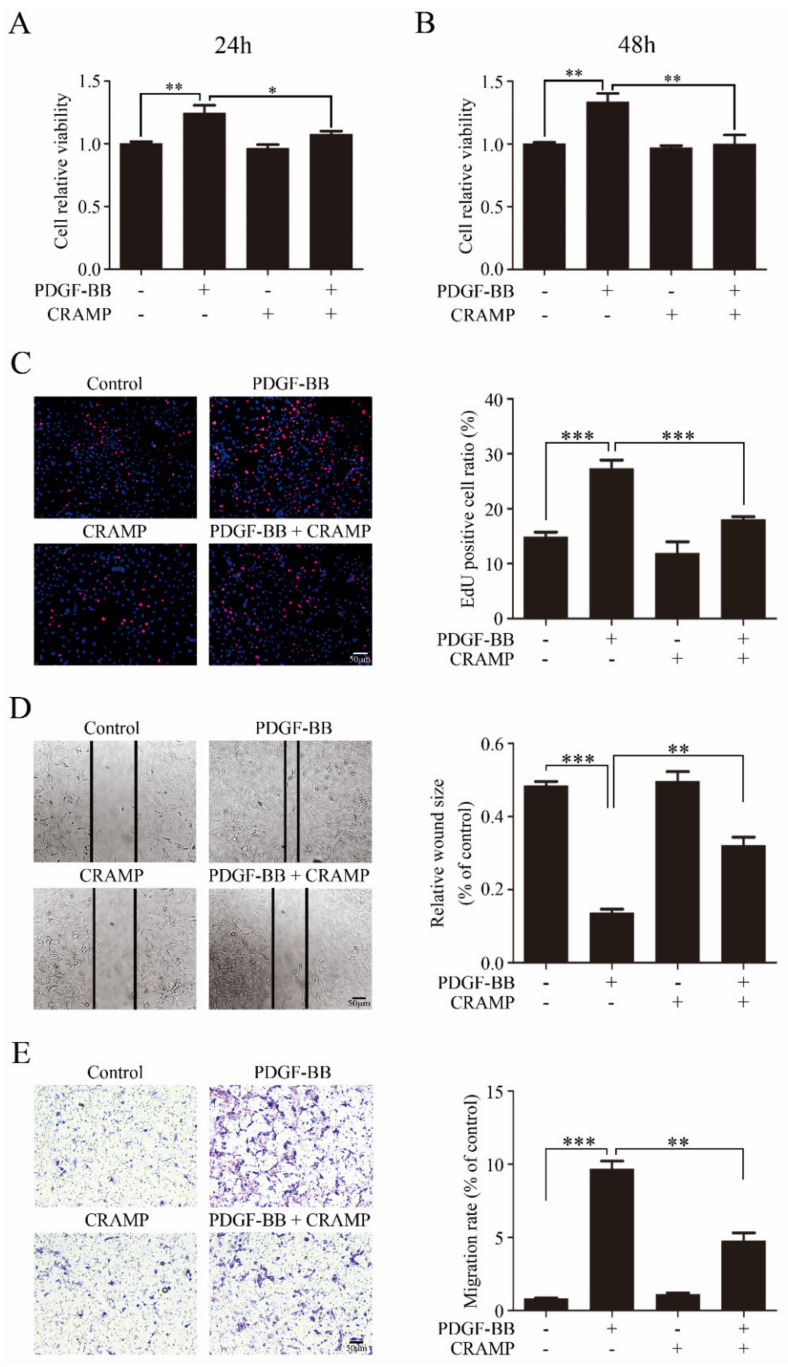
CRAMP inhibits PDGF-BB elevated VSMC proliferation and migration. (**A**,**B**) VSMC were pretreated with CRAMP (100 ng/mL) for 2 h and then stimulated with PDGF-BB (20 ng/mL) for 24 h or 48 h. Cell viability was detected using 3-(4, 5-dimethylthiazol-2-yl)-2, 5-diphenyl tetrazolium bromide (MTT) assay. Data of 3 independent experiments is presented as mean ± SEM. ** *p* < 0.01 compared with control, *n* = 8. (**C**) VSMC were pretreated with CRAMP (100 ng/mL) for 2 h and then stimulated with PDGF-BB (20 ng/mL) for 24 h. Proliferation of VSMC was detected using EdU assay. Data of 3 independent experiments is presented as mean ± SEM. *** *p* < 0.001 compared with control, *n* = 6. (**D**,**E**) VSMC were pretreated with CRAMP (100 ng/mL) for 2 h and then stimulated with PDGF-BB (20 ng/mL) for 24 h. Proliferation of VSMC was detected using wound healing assay (**D**) and transwell assay (**E**). Data of 3 independent experiments is presented as mean ± SEM. ** *p* < 0.01, *** *p* < 0.001 compared with control, *n* = 3.

We then detected the effects of CRAMP on VSMC proliferation and migration. As showed in Figure 2A,B CRAMP significantly inhibited PDGF-BB-enhanced cell viability of VSMC. The EdU assay also showed that CRAMP could decrease PDGF-BB-mediated VSMC proliferation. Followingly, we detected the wound healing assay and transwell assay, and the results showed that CRAMP could significantly inhibit both PDGF-BB-induced VSMC migration and invasion. Above data suggested that CRAMP could inhibit PDGF-BB-elevated VSMC proliferation and migration.

### 3.2. CRAMP Inhibited PDGF-Mediated IL-6/STAT3 Activation

**Figure 3:**
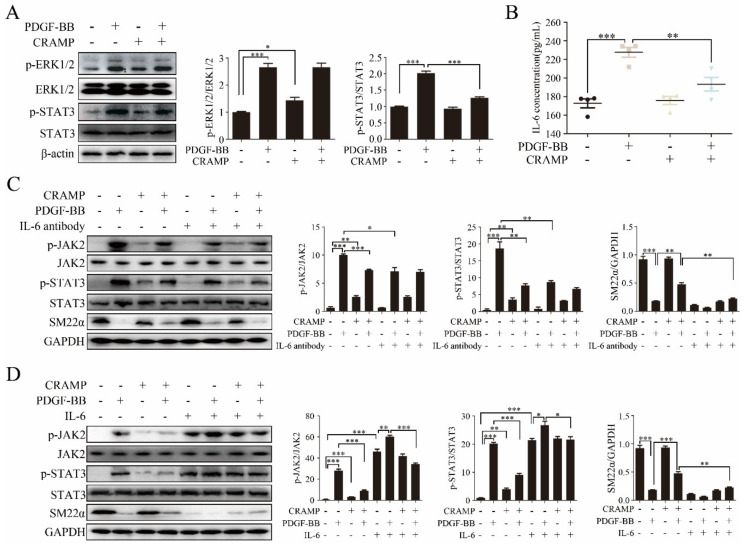
IL-6/JAK2/STAT3 cascades participated in CRAMP inhibited VSMC Phenotypic transformation. (**A**) VSMC were pretreated with CRAMP (100 ng/mL) for 2 h and then stimulated with PDGF-BB (20 ng/mL) for 24 h. The activation of ERK1/2 and STAT3 was detected using immunoblotting with anti-p-ERK1/2, anti-STAT3 antibodies. Data of 3 independent experiments is presented as mean ± SEM. * *p* < 0.05, *** *p* < 0.001 compared with control, *n* = 3. (**B**) VSMC were pretreated with CRAMP (100 ng/mL) for 2 h and then stimulated with PDGF-BB (20 ng/mL) for 24 h. IL-6 in culture medium was detected by enzyme-linked immunosorbent assay (ELISA). Data of 3 independent experiments is presented as mean ± SEM. *** *p* < 0.001 compared with control, *n* = 4. (**C**) VSMC were pretreated with IL-6 antibody (10 μg/mL) for 2 h and CRAMP (100 ng/mL) for 2 h and then stimulated with PDGF-BB (20 ng/mL) for 24 h followed by immunostaining with p-JAK2, p-STAT3 and SM22α antibodies. (**D**) VSMC were pretreated with recombinant IL-6 at final concentration of 10 ng/mL and CRAMP (100 ng/mL) for 2 h and then stimulated with PDGF-BB (20 ng/mL) for 24 h followed by immunostaining with p-JAK2, p-STAT3 and SM22α antibodies. Data of 3 independent experiments is presented as mean ± SEM. ** *p* < 0.01, *** *p* < 0.001 compared with control, *n* = 3.

Activation of ERK1/2 and STAT3 plays an effective role in VSMC phenotypic switching [30,31,32,33,34,35]. To find out the mechanisms of CRAMP in regulating VSMC phenotypic modulation, we first examined the effects of CRAMP on ERK1/2 and STAT3 activation. As showed in Figure 3A, the phosphorylation of ERK1/2 and STAT3 were significantly enhanced when treated with PDGF-BB, while the level of p-STAT3 but not p-ERK1/2 was inhibited when treated with both PDGF-BB and CRAMP.

**Figure 4:**
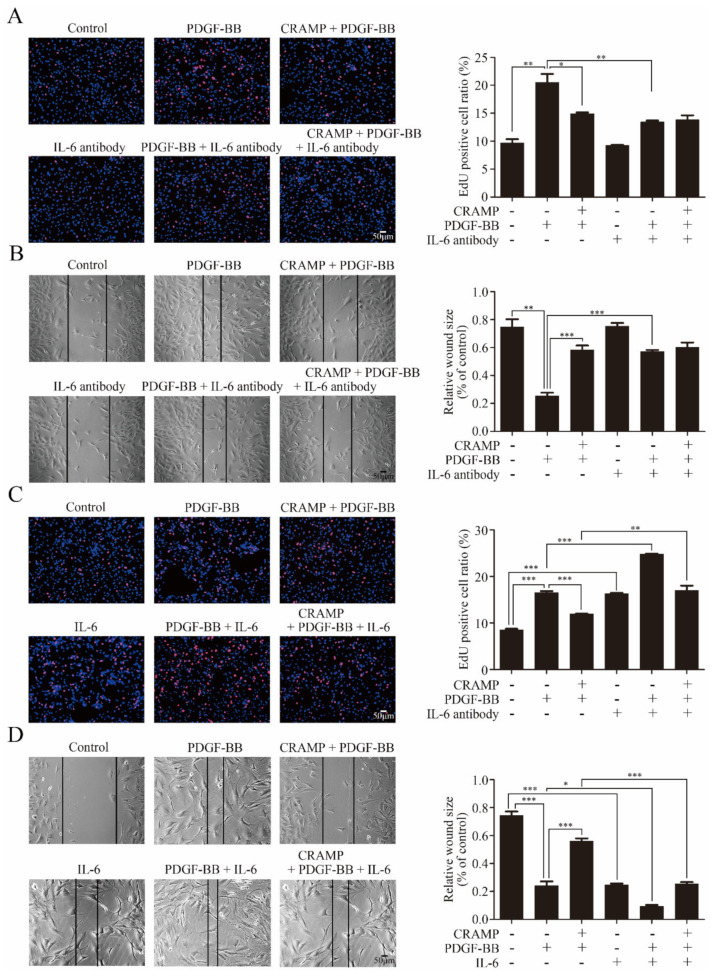
IL-6/JAK2/STAT3 cascades participated in CRAMP inhibited VSMC proliferation and migration. (**A**) VSMC were pretreated with IL-6 antibody (10 μg/mL) for 2 h and CRAMP (100 ng/mL) for 2 h and then stimulated with PDGF-BB (20 ng/mL) for 24 h. The proliferation of VSMC was detected by EdU assay. (**B**) VSMC were pretreated with IL-6 antibody (10 μg/mL) for 2 h and CRAMP (100 ng/mL) for 2 h and then stimulated with PDGF-BB (20 ng/mL) for 24 h. The migration of VSMC was detected by wound healing assay. (**C**) VSMC were pretreated with IL-6 (10 ng/mL) for 2 h and CRAMP (100 ng/mL) for 2 h and then stimulated with PDGF-BB (20 ng/mL) for 24 h. The proliferation of VSMC was detected by EdU assay. (**D**) VSMC were pretreated with recombinant IL-6 at final concentration of 10 ng/mL and CRAMP (100 ng/mL) for 2 h and then stimulated with PDGF-BB (20 ng/mL) for 24 h. Data of 3 independent experiments is presented as mean ± SEM. * *p* < 0.05, ** *p* < 0.01, *** *p* < 0.001 compared with control, *n* = 4.

As IL-6 was a classical activator of JAK2-STAT3 signaling [36], we hypothesized that CRAMP inhibited PDGF-BB-mediated VSMC phenotypic transformation by modulating autocrine IL-6. As shown in Figure 3B, IL-6 level was significantly increased with the treatment of PDGF-BB while this enhancement was blocked when added with CRAMP-treatment. As shown in Figure 3C, IL-6 neutralized antibody could significantly inhibit the activation of JAK2-STAT3 pathway and block PDGF-BB-induced decrease of SM22α. Conversely, exogenous IL-6 activated the JAK2-STAT3 pathway and reduced SM22α expression in VSMC and reduced the role of CRAMP on VSMC (Figure 3D). Similarly, blocking IL-6 significantly inhibited PDGF-BB-induced VSMC proliferation and migration (Figure 4A,B). Exogenous IL-6 promoted the proliferation and migration of VSMC and partially attenuated the role of CRAMP (Figure 4C,D). All these results suggested that CRAMP regulated JAK2/STAT3 cascade through blocking IL-6 autocrine production.

### 3.3. CRAMP Regulated IL-6 Autocrine via Targeting NF-κB Signaling

**Figure 5:**
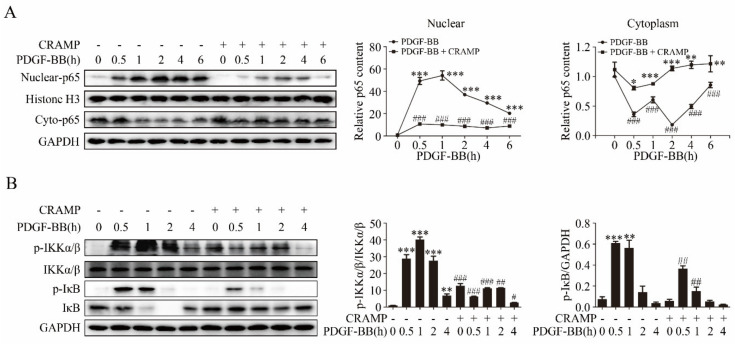
CRAMP regulated IL-6 autocrine via targeting NF-κB signaling. (**A**) VSMC were pretreated with CRAMP (100 ng/mL) for 2 h and then stimulated with PDGF-BB (20 ng/mL) at different times (0.5, 1, 2, 4, 6 h). The nuclear- and cyto-p65 were detected by immunoblotting. (**B**) VSMC were pretreated with CRAMP (100 ng/mL) for 2 h and then stimulated with PDGF-BB (20 ng/mL) at different times (0.5, 1, 2, 4, 6 h). The activation of inhibitor of nuclear factor kappa-B kinase (IKKα/β) was detected by immunoblotting. Data of 3 independent experiments is presented as mean ± SEM. * *p* < 0.05, ** *p* < 0.01, *** *p* < 0.001 compared with control; ^#^ *p* < 0.05, ^##^ *p* < 0.01, ^###^ *p* < 0.001 compared with PDGF-BB treatment group, *n* = 3.

NF-κB is an important transcription factor regulating IL-6 expression [37,38] and plays a critical signaling in VSMC dedifferentiation, proliferation and migration [39,40]. We then investigated whether CRAMP regulates the autocrine production of IL-6 through targeting NF-κB. As showed in Figure 5A, PDGF-BB significantly promoted the nuclear translocation of NF-κB p65 and CRAMP could effectively inhibit it. Following, we detected the activation of IKKα/β and IκB, upstream of p65, the western blot results showed that CRAMP also blocks the activation of IKKα/β and IκB by PDGF-BB (Figure 5B). These results indicated that CRAMP regulates the autocrine of IL-6 by targeting the NF-κB pathway.

### 3.4. CRAMP Prevented PDGF-BB-Enhanced ROS by Targeting NOX1

**Figure 6:**
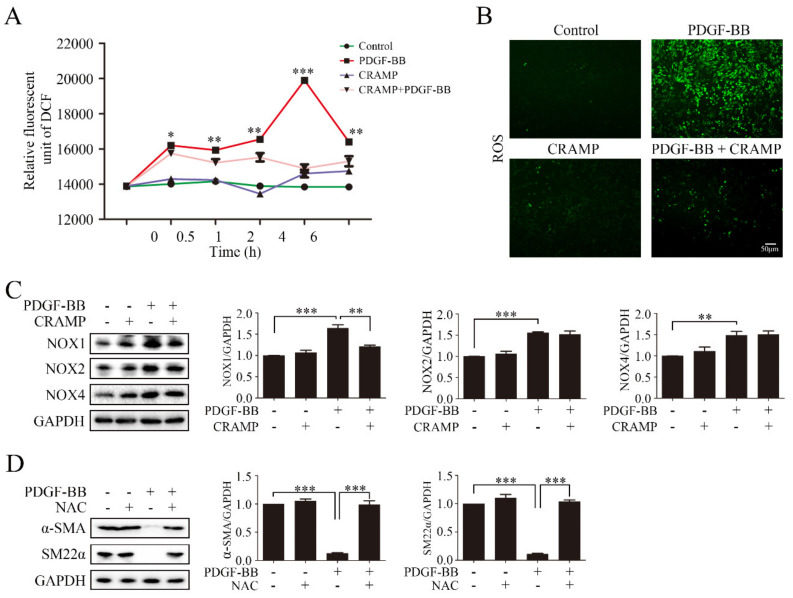
CRAMP prevented PDGF-BB-enhanced ROS by targeting NOX1. (**A**) VSMC were pretreated with CRAMP (100 ng/mL) for 2 h and then stimulated with PDGF-BB (20 ng/mL) at different times (0.5, 1, 2, 4, 6 h). The level of ROS was detected using fluorescence microplate reader. Data of 3 independent experiments is presented as mean ± SEM. * *p* < 0.05, ** *p* < 0.01, *** *p* < 0.001 compared with control, *n* = 5. (**B**) VSMC were pretreated with CRAMP (100 ng/mL) for 2 h and then stimulated with PDGF-BB (20 ng/mL) for 4 h. The level of ROS was detected by immunofluorescence detection. (**C**) VSMC were pretreated with CRAMP (100 ng/mL) for 2 h and then stimulated with PDGF-BB (20 ng/mL) for 24 h following by immunoblotting with anti-NOX1, anti-NOX2 and anti-NOX4 antibodies. (**D**) VSMC were pretreated with CRAMP (100 ng/mL) for 2 h and then stimulated with PDGF-BB (20 ng/mL) for 24 h followed by immunoblotting with anti-α-SMA and anti-SM22α antibody. Data of 3 independent experiments is presented as mean ± SEM. * *p* < 0.05, ** *p* < 0.01, *** *p* < 0.001 compared with control, *n* = 3.

Intracellular ROS accumulation is critical for NF-κB activation [37]. Our results showed that pretreated with CRAMP inhibited PDGF-BB could significantly increase the ROS level, and CRAMP alone could also enhance the ROS level after 4 h, but at the same time, CRAMP could inhibit the effect of PDGF-BB (Figure 6A,B). Since CRAMP can affect ROS levels in VSMC, we wonder whether CRAMP plays a role in major ROS producing enzyme NADPH oxidase (NOX). The results showed that the protein abundance of NOX1, NOX2 and NOX4 increased in PDGF-BB treated cells. CRAMP can significantly inhibit the increase of NOX1 induced by PDGF-BB, but has no significant effect on NOX2 and NOX4 (Figure 6C). Next, we used ROS scavenger N-acetyl-L-cysteine (NAC) to confirm the role of NOX1-ROS in PDGF-BB-mediated phenotypic transformation, as showed in Figure 6D, NAC could significantly reverse the reduction of α-SMA and SM22α which induced by PDGF-BB. These results indicate that CRAMP can inhibit PDGF-BB function by regulating ROS levels in VSMC and targeting NOX1.

### 3.5. CRAMP Repressed Intimal Hyperplasia and Suppressed ROS/IL-6 Generation In Vivo

**Figure 7:**
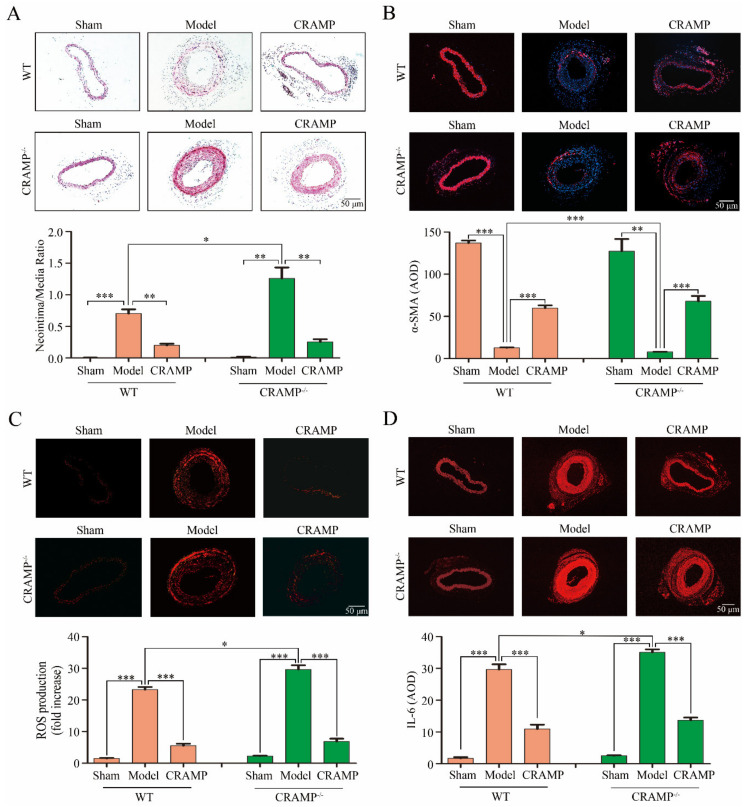
CRAMP repressed intimal hyperplasia and suppressed ROS/IL-6 generation in vivo. (**A**) Representative hematoxylin-eosin (HE) staining of carotid arteries in sham and injury rats. (**B**) Representative CD45 SM22α staining of carotid arteries from WT and CRAMP^−/−^ mouse. Red fluorescence: α-SMA, Blue: nuclear. (**C**) In situ dihydroethidium (DHE) staining of mouse carotid arteries. Red fluorescence: ROS. (**D**) Representative IL-6 staining of carotid arteries from WT and CRAMP^−/−^ mouse. Red fluorescence: IL-6. Data is presented as mean ± SEM. * *p* < 0.05, ** *p* < 0.01, *** *p* < 0.001 compared with control, *n* = 8.

Compared with the Sham group, significant intimal hyperplasia was observed both in the wild type (WT) and CRAMP knockout (CRAMP^−/−^) mice, and the proportion of intimal hyperplasia in the CRAMP^−/−^ mice was higher than that in the WT mice (Figure 7A). Administration of CRAMP obviously reduced the ratio of neointimal area to media area in injured carotid arteries of both WT and CRAMP^−/−^ mice (Figure 7A). The phenotypic transformation marker α-SMA are significantly reduced in both WT and CRAMP^−/−^ model groups, and CRAMP can significantly inhibit this phenomenon (Figure 7B). Meanwhile, the ROS level and IL-6 level were also significantly enhanced in both WT and CRAMP^−/−^ model groups, and CRAMP could significantly block it (Figure 7C,D). All these results demonstrated that CRAMP repressed intimal hyperplasia and suppressed ROS/IL-6 generation.

## 4. Discussion

Atherosclerosis is a chronic progressive inflammatory disease and a leading cause of death worldwide [41,42,43]. In the samples of atherosclerosis patients, the content of LL-37 (CRAMP in mice) was much higher than that of normal volunteers [44], suggesting that LL-37 may play a role in the development of atherosclerosis. In present study, we explored the role of CRAMP in regulating the phenotype transformation and the underlying mechanism of VSMC exposed to PDGF-BB. Our results demonstrated that CRAMP treatment blocked NF-κB nuclear translocation, IL-6 autocrine and thus the expression of phenotypic transformation marker, α-SMA and SM22α in VSMC. Collectively these findings demonstrated a role in attenuated phenotype transformation, proliferation and migration of VSMC in response to PDGF-BB. Results from the present study also suggested that CRAMP participate oxidative stress, inhibiting ROS and following regulating NF-κB/IL-6 cascade. Additional studies will be required to test the direct target of CRAMP and to address the role of CRAMP for the regulation of ROS production in response to other stimuli.

IL-6 is a pleiotropic cytokine. Several studies indicated that IL-6 has critical pathophysiological roles in cardiovascular diseases, such as atherosclerosis [45,46]. Nevertheless, it has been suggested that locally secreted IL-6 is involved in the VSMC proliferation in response to PDGF [47]. Similarly, our results showed that under the stimulation of PDGF-BB, VSMC can secrete IL-6, which can regulate the phenotypic transformation of VSMC, and this process is realized by CRAMP by regulating oxidative stress, that is, inhibiting the production of ROS.

Previous studies have demonstrated that incubation of VSMC with PDGF-BB triggers the rat sarcoma (Ras)/ rapidly accelerated fibrosarcoma (Raf)/ mitogen-activated protein kinase kinase (MEK)/ extracellular regulated protein kinases (ERK) kinase cascade, which leads to Elk-1 phosphorylation and the inhibition of SMC, specific marker genes, via the displacement of myocardin from smooth muscle cell-specific prompters [48]. Our present results showed that PDGF-BB enhanced the phosphorylation of ERK1/2 but CRAMP failed to block the activation although it has the similar effects with PDGF-BB all alone. Additional studies will be needed to unravel whether CRAMP has functions on other mitogen-activated protein kinases including p38 and JNK, and how CRAMP exerts functions on VSMC alone. Furthermore, accumulating evidence supports an important role for the activation of STAT3 in PDGF-BB-induced VSMC proliferation and migration [49]. Similarly, our results demonstrated that PDGF-BB regulates VSMC phenotypic transformation through the IL-6-STAT3 pathway and CRAMP could block the activation.

The NOX catalytic subunits-NOX1, NOX2, and NOX4-share a conserved structure and associate with p22phox on the cell membranes and are expressed in the vascular wall cells in rodents and humans [50]. NOX1 is predominantly expressed in VSMC and NOX1 plays a critical role in VSMC function in response to pathophysiological stimuli. The NOX1 expression is induced in neointimal SMC after vascular injury where it mediates cell migration, proliferation, and extracellular matrix production, while NOX1 deletion significantly reduces neointima hyperplasia [51,52,53]. The NF-κB pathway is the main pathway through which NOX1 plays its role, while ROS are often considered as the second messenger to mediate the activation of NF-κB [54,55]. The activation of NF-κB by ROS, specifically ROS generated by NOX, has been shown in VSMCs and other cells, and induced IL-6 release [56,57]. There have been many reports about the relationship between CRAMP/LL-37 and ROS. CRAMP/LL-37 can regulate the immune response by increasing the production of NOS and ROS during the treatment of infection [58]. LL-37 also increased platelet–neutrophil aggregates formation and activated neutrophil through promoting the production of ROS [59]. Recent studies showed that CWA was able to reduce LPS-induced ROS accumulation both in macrophages and in mice [27] and effectively decreased the level of ROS in E. coli K88-induced macrophages [26]. The results above indicated that the regulatory effect of CRAMP on ROS varies according to different states. Our current results also indicated that CRAMP can effectively inhibit ROS production induced by PDGF-BB. The relationship between CRAMP and ROS under other conditions remains to be further studied.

Herein, we have demonstrated that CRAMP modulates phenotypic transformation in VSMC. One potential mechanism by which this occurs is via CRAMP/autocrine IL-6/JAK2/STAT3 cascade; However, we also provided evidence that CRAMP regulates ROS and ROS-mediated NF-κB activation. These data suggest critical mechanism by which CRAMP regulates oxidation stress and cell differentiation. Given the reported relevance of CRAMP in atherosclerosis, the present findings which demonstrate a strong impact of CRAMP on the activation of ROS/NF-κB/IL-6 studies and provide new insights into the mechanisms by which CRAMP can regulate oxidation signaling in cardiovascular diseases like atherosclerosis.
